# Features of Vat-Photopolymerized Masters for Microfluidic Device Manufacturing

**DOI:** 10.3390/bioengineering11010080

**Published:** 2024-01-15

**Authors:** Maria Laura Gatto, Paolo Mengucci, Monica Mattioli-Belmonte, Daniel Munteanu, Roberto Nasini, Emanuele Tognoli, Lucia Denti, Andrea Gatto

**Affiliations:** 1 Department of Industrial Engineering and Mathematical Sciences (DIISM), Università Politecnica delle Marche, Via Brecce Bianche 12, 60131 Ancona, Italy; m.l.gatto@staff.univpm.it; 2 Department of Materials, Environmental Sciences and Urban Planning (SIMAU), Università Politecnica delle Marche, Via Brecce Bianche 12, 60131 Ancona, Italy; p.mengucci@staff.univpm.it; 3 Department of Clinical and Molecular Sciences, Università Politecnica delle Marche, Via Tronto 10/a, 60126 Ancona, Italy; m.mattioli@staff.univpm.it; 4 INSTM, National Consortium of Materials Science and Technology, Via G. Giusti 9, 50121 Florence, Italy; 5 Material Science Department, Transilvania University of Brasov, 29 Eroilor Blvd., 500036 Brasov, Romania; danielmunteanu@unitbv.ro; 6 Prosilas S.r.l., Via Terracini 14, 60212 Civitanova Marche, Italy; 7 Department of Engineering “Enzo Ferrari”, Università di Modena e Reggio Emilia, Via P. Vivarelli 10, 41125 Modena, Italy; lucia.denti@unimore.it (L.D.); agatto@unimore.it (A.G.)

**Keywords:** microfluidic device, additive manufacturing, vat photopolymerization, benchmark master, PDMS replica

## Abstract

The growing interest in advancing microfluidic devices for manipulating fluids within micrometer-scale channels has prompted a shift in manufacturing practices, moving from single-component production to medium-size batches. This transition arises due to the impracticality of lab-scale manufacturing methods in accommodating the increased demand. This experimental study focuses on the design of master benchmarks 1–5, taking into consideration critical parameters such as rib width, height, and the relative width-to-height ratio. Notably, benchmarks 4 and 5 featured ribs that were strategically connected to the inlet, outlet, and reaction chamber of the master, enhancing their utility for subsequent replica production. Vat photopolymerization was employed for the fabrication of benchmarks 1–5, while replicas of benchmarks 4 and 5 were generated through polydimethylsiloxane casting. Dimensional investigations of the ribs and channels in both the master benchmarks and replicas were conducted using an optical technique validated through readability analysis based on the Michelson global contrast index. The primary goal was to evaluate the potential applicability of vat photopolymerization technology for efficiently producing microfluidic devices through a streamlined production process. Results indicate that the combination of vat photopolymerization followed by replication is well suited for achieving a minimum rib size of 25 µm in width and an aspect ratio of 1:12 for the master benchmark.

## 1. Introduction

Microfluidics involves the manipulation and analysis of fluids confined within micrometer-scale channels, achieved through microfabrication techniques [1,2]. The main purpose of microfluidics research is to integrate various components for fluid manipulation, such as pumps, valves, filters, and mixers, along with analytical separation and detection techniques. This integration aims to create a single device capable of comprehensive on-chip analysis [3,4]. Presently, microfluidics has proven successful in various biological applications, including DNA sequencing, PCR amplification, analysis of amino acids, peptides, and proteins, and immunoassays, as well as the handling and sorting of cells and in vitro fertilization [3,5,6].

The initial step in designing a microfluidic device involves selecting the shear rate value, followed by determining the aspect ratio (width/height) that defines the channel dimensions. A low width/height ratio (e.g., 4:1) minimizes fluid volume but results in a non-uniform shear rate across the channel width. Conversely, a high width/height ratio (e.g., 10:1) ensures a more uniform shear rate profile but requires a larger fluid volume [7]. The literature presents numerous designs of microfluidic devices. For instance, studies on blood rheology [8] reveal that blood behaves as a Newtonian fluid at shear rates exceeding 100 s^−1^. Authors like Shul’man et al. [9] investigated rectangular microchannels with aspect ratios of 4:1 and 10:1, with a fixed height of 50 µm, ensuring shear rates of 1000 s^−1^ and 100 s^−1^, respectively. Other studies report the width of rectangular channels in the range of 10 to 500 µm [10,11,12]. Consequently, there is no standard reference for microchannel dimensions, resulting in diverse custom approaches. For example, Nalayanda et al. [6] explored a device with 10–1000 mm wide linear channels spaced 50–100 mm apart, while Sarvepalli et al. [11] compared the performance of a microfluidic chamber with a 500 × 50 mm^2^ microchannel section to that of a standard parallel plate chamber (6000 × 127 mm^2^). Additional flow channel dimensions reported in the literature include 200 × 24 µm^2^ [10] and 100 × 80 µm^2^ [13].

Polydimethylsiloxane (PDMS) is currently the polymer commonly used in fabricating microfluidic devices due to its cost-effectiveness, biocompatibility, and transparency, making it suitable for optical detection [5,14,15]. Furthermore, PDMS surfaces are inert and non-reactive with many reagents, and PDMS cures at relatively low temperatures and is gas-permeable [16]. However, PDMS does have some drawbacks, such as non-specific adsorption of biological and chemical analytes and high hydrophobicity [5].

A substantial portion of research in the realm of PDMS-based microfluidic devices has been conducted using soft lithography, a technique originally introduced by G. Whitesides in 1998 [17]. The production of a PDMS stamp using soft lithography, also known as the replica molding technique [18], involves four main steps: (1) Deposition of a negative photoresist (a photo-active polymer) on a substrate, which is then exposed to UV light through a carefully designed mask. The unexposed photoresist is dissolved, leaving the cured photoresist on the substrate with the pattern outlined by the mask. This resulting structure is called a master; (2) subjecting the master to a chemical treatment to minimize its adherence to the stamp; (3) pouring PDMS over the master and curing it, for allowing the PDMS stamp to be peeled off the master; and (4) fabrication of micro- and nanostructures with the stamp by printing, molding, and embossing [14,19].

Various lithography techniques, such as reactive-ion etching, electron-beam lithography, wet etching, multiphoton lithography, direct laser writing, and focused ion beam, have been used to fabricate master molds. These methods enable high-resolution fabrication, and some, being software-controlled, do not necessitate the use of masks (e.g., direct laser writing, electron beam, and focused-ion beam). This characteristic holds the potential for cost reduction. However, it is essential to note that these processes are typically low speed, posing limitations on the commercialization pathway [20].

The soft lithography technique for PDMS microdevices offers several advantages, including low-cost processing, rapid prototyping, ease of design, direct molding and sealing of devices, reusability of masters, and applicability to various biological processes due to the polymer’s biocompatibility [3,14]. However, despite the success of PDMS-based microfluidic devices produced by soft lithography, some significant disadvantages exist.

PMDS devices are often not rugged, leading to potential flow profile issues due to leakage and/or uneven pressure. The fabrication efficiency of lithography is low, involving multi-step processes unsuitable for medium batch size production [21] for direct industrial implementation [22]. Moreover, adjusting device features quickly is challenging without creating a new master, and the master may collapse or bend if the width-to-height ratio is less than 1:4. Even if the master is robust enough to avoid bending, extraction from the cast PDMS without damage becomes complex if the width-to-height ratio is less than 1:10. The maximum achievable height for lithographed features is 150 µm, and the walls are constrained to be vertical, resulting in a rectangular cross-section. UV diffraction during the curing phase can cause adjacent features to stick in undesirable zones, and the replication step is only suitable for channels with a width-to-height ratio of less than 10:1, as there is a risk of collapse in the bridge between beams in the replica [23]. Additionally, while PDMS devices with integrated functional parts have been reported, they typically involve the integration of only two discrete devices [14,16].

Additive manufacturing (AM) has recently emerged as an alternative fabrication method for creating acrylic resin-based masters or direct components for microfluidics, facilitating the production of intricate parts. For example, in the production of a PDMS replica, an AM master can include reference geometries for subsequent assembly and lateral mold walls. Moreover, AM techniques exhibit the potential to streamline the labor-intensive and multi-step lithography process associated with PDMS-based device production [16,24,25,26]. For instance, AM-based solutions eliminate conventional steps, such as laser cutting of the acrylic mold needed for PDMS casting [27].

While the minimum size of additively manufactured structures is larger than that achievable by soft lithography—for example, vat photopolymerization (VPP) allows fabricating details smaller than 100 μm [28,29] compared to the 1 μm achievable by soft lithography [30]—the flexibility and short lead time of AM processes compensate for this disadvantage when high resolution is not imperative [31]. Furthermore, soft lithography can achieve geometrical height values exceeding ≈ 50–100 μm, but it involves the deposition and baking of multiple photoresist layers, each with a distinct photomask, prior to patterning. AM techniques effectively surmount this limitation [22,32,33,34].

However, not all AM techniques are suitable for microfluidic fabrications. Microfluidic devices must meet essential criteria for proper operation, including flexibility, biocompatibility, precise design and geometry, optical transparency, and reliability of microchannels. Only a few polymeric AM techniques align with these requirements [5,35,36].

VPP emerges as an ideal technology for microfluidic device fabrication, offering high printing resolution and accuracy, faster printing times, and greater flexibility in material tailoring and development compared to other polymeric AM techniques [5,17]. VPP-printed microdevices can easily integrate standard connectors for fluid sources, facilitating plug-and-play functionality. Additionally, the process of flushing unpolymerized resin from micro-voids after VPP is considerably simpler than the removal of solid sacrificial support required by other 3D printing processes such as material jetting and material extrusion [37,38]. VPP relies on the selective polymerization of photopolymers using a laser beam focused on a liquid photopolymer in a vat. Subsequent post-curing processes are applied to achieve the highest chemical conversions [5].

Biological performances of additively manufactured microfluidic devices have been extensively explored in the literature; however, a notable knowledge gap exists regarding the applicability and performance of AM solutions within the microfluidic field [35], particularly concerning constraints and expected outcomes.

This experimental study addresses this gap by designing benchmark masters with the aim of simplifying the manufacturing cycle and reducing certain steps associated with conventional techniques. The objective is to significantly impact the time-to-market required for producing PDMS microfluidic devices. Three distinct benchmark masters (BM1, BM2, and BM3) were specifically designed to assess the feasibility and dimensional limits of ribs produced by VPP, employing a set of process parameters and a material that has been previously optimized and described by the authors [39]. Building upon the insights gained from BM1–BM3, two additional benchmark masters (BM4 and BM5) were designed for applications in the microfluidic field. For this purpose, PDMS replicas of BM4 and BM5 were fabricated by casting.

In addition to the masters, the analysis of the replicas was a crucial aspect for validating the entire production process and guiding biotechnologists’ decisions for production improvement, especially in the presence of significant variability in device dimensions and shapes.

Following the VPP fabrication of the masters and PDMS casting of the replicas, a comprehensive study of the ribs and channels’ dimensions, in terms of width, height, depth and width/height ratio, was conducted. The obtained results aim to furnish experimental data on the dimensional tolerances of the master benchmarks fabricated with VPP and on the dimensional deviations between the master benchmarks and the corresponding replicas produced with PDMS. This information is valuable for advancing the development of devices in the field of microfluidics.

The aim of this experimental work was to provide knowledge on the microfluidic device manufacturing process, focusing on the dimensional properties of benchmark masters produced by VPP and the replicas of the devices obtained by PDMS casting.

## 2. Materials and Methods

### 2.1. Design of Benchmark Masters

Five different benchmark masters were designed with geometries illustrated in Table 1. The geometrical features considered in this study include ribs, required for creating channels in the microfluidic part, and the inlet, outlet, and reaction chamber, which allow for the connection of the ribs in the master and, consequently, the connection of the channels in the replica. Thus, the parameters considered for the design were the rib width (µm) and height (µm) and the relative width/height ratio.

Benchmark 1 (BM1) and benchmark 2 (BM2) integrate the master mold and casting box into a single part. Both BM1 and BM2 present 4 ribs. Specifically, BM1 ribs were designed with constant width/height ratio (1:8), while the ribs of BM2 have a constant height of 3 mm and, as a consequence, different width/height ratios (Table 1). Benchmark 3 (BM3) partially reproduces the ribs of BM2 in terms of width (Table 1) but was designed with a separate casting box to evaluate the effects of sidewalls on the accuracy of ribs built on the removable base. Furthermore, the casting box allows a better removal of PDMS replicas from the master base produced by VPP. In benchmark 4 (BM4) and benchmark 5 (BM5), the ribs were connected to the inlet, outlet, and the reaction chamber of the master, useful for replica production (Table 1). Specifically, BM4 and BM5 were designed for creating a microfluidic device with four and three reaction chambers, respectively. Moreover, in BM4 the width/height ratio ranges from 1.3:1 to 10:1, while in BM5 the width/height ratio is fixed at 1:1 (Table 1). Additionally, based on the results of the accuracy of ribs obtained for BM3, BM4 and BM5 were designed with a removable casting box, for microfluidic applications.

### 2.2. Benchmark Master Material

All benchmark masters were produced by the VPP process using an acrylic acid ester commercially known as VITRA DL375 (DWS, Thiene, Italy), as detailed in a previous paper by the authors [39] (VITRA DL375 specifications are listed in the Supplementary Materials [40]).

### 2.3. Manufacturing of the Benchmarks

#### 2.3.1. Manufacturing of Benchmark Masters

Benchmark (BM) masters were fabricated using a DWS 029X manufacturing system (DWS, Thiene, Italy). The BMs were oriented with the base surface parallel to the build platform of the AM machine. The parameters optimized for the VPP process are listed in Table 2. These process parameters were described in a previous work by the authors [39] and were based on industrial know-how.

The production of the benchmark masters by VPP involved the following post-processing steps:

- Washing in 96% ethyl alcohol using compressed air to eliminate unpolymerized resin from the as-built part;
- Further ultrasonic washing in ethyl alcohol to ensure the removal of unpolymerized resin from the channels and cavities;
- Post-curing in UV oven for 20 min;
- Supports removal and surface finishing.

#### 2.3.2. Manufacturing of Benchmark Replicas

Replicas of BM4 and BM5 were fabricated using a standard procedure for casting PDMS in a master mold (the PDMS properties are listed in the Supplementary Materials [41]). The production of the replicas by PDMS casting included the following steps:

- Mixing two-component silicone (PDMS resin to curing agent mix ratio: 10:1);
- 1° degassing of the silicone under vacuum (approx. 1 h);
- Pouring into the master mold fabricated by VPP;
- 2° degassing of the silicone under vacuum in the mold (approx. 1 h);
- Curing at room temperature (approx. 48 h);
- Removal from the mold;
- Deburring, if necessary.

The overall fabrication time for the entire process is approx. 52–53 h.

### 2.4. Characterization of the Benchmarks

#### 2.4.1. Methodology Validation

To determine the width and height of the ribs of BM1, BM2, and BM3 masters, a preliminary non-destructive analysis was carried out to evaluate the measurement error of two different instruments. The Nikon SMZ1270 optical stereo microscope (Nikon, Tokyo, Japan) was used to perform optical microscopy analysis, while the Nikon Eclipse LV150N optical microscope (Nikon, Tokyo, Japan), equipped with a confocal head, was used for profilometry analysis. The size of the features to be analyzed was within the measurement range of both instruments, but optical microscopy analysis requires significantly less time than the profilometry analysis. The Nikon SMZ1270 was employed to measure the height of the ribs under the optical microscope, taking three measurements for each rib. Using the Nikon Eclipse LV150N microscope, the height of the ribs was determined from the profilometry analysis by evaluating the 3D point data, which was post-processed with the Mountains Map software (Digital Surf, Besançon, France). The post-processing included a “remove form” operator with a first-degree polynomial to make the surface planar, and a “remove outliers” operator to remove incorrect measurement values. For both optical microscopes, three measurements were taken on each rib, providing mean values, corresponding standard deviations, and the percentage error from the nominal value.

BM3 was used for validating the measurements obtained by the two aforementioned instruments, given the separability between the removable base and the walls of the casting box, which facilitates the analysis procedures.

Furthermore, a test was planned to assess the loss of efficiency when observing with an optical microscope through a photopolymerized wall. The test was performed on BM3, where the casting box was built separately from the removable base. The fabrication of the separate casting box makes the device a hybrid solution, where some components are produced directly, such as the walls, and others are replicated by PDMS. Casting PDMS in an assembled master consisting of a lid and a separate open box provides the option to remove or retain the side walls. The optical properties are critical for a flexible solution where the box is built separately from the lid and does not need to be removed. The walls that are not removed must provide readability of the cells in the device chamber by optical microscopy, as well as maintain the overall dimensional tolerances. Quantification of the optical signal is crucial for deciding whether the physical confining edge of the PDMS melt can be left in place. Thus, readability analysis allows evaluating the loss of efficiency when observed with an optical microscope, through a photopolymerized wall. For this purpose, a laser-marked microscope calibration ruler was observed both directly and through a 1 mm-thick photopolymerized wall under identical illumination conditions using a WILD M3Z macroscope (Leica GmbH, Wetzlar, Germany) with a camera. A method was proposed to evaluate the possible attenuation of contrast due to the presence of the resin wall, following the approach in [42].

A grayscale image can be represented by a matrix X (m, n). Each pixel X_i,j_ varies in the range of values [0; 2k−1], where k is the number of bits encoding the pixel. An image with 200 × 200 pixels can be converted to a 200 × 200 matrix in which each element ranges between 0 (darkest pixel, absolute shadow) and 255 (brightest pixel, absolute light). A number of factors and numerical variables contribute to the realization of the digital image, all of which must be controlled. Several formulas have been proposed in the literature for evaluating the global contrast (*C*) of a digital image, e.g., the Michelson formula in Equation (1) for periodic patterns [42]:

(1) $$C=\frac{L_{max}-L_{min}}{L_{max}+L_{min}}$$

where *L_max_* and *L_min_* represent the maximum and minimum luminance, respectively. The test consisted of using a 144-led annular light source at 3000–40,000 lx, T = 6400 K. A series of preliminary tests guided the selection of illumination conditions and exposure time for ruler acquisition by direct observation and through the photopolymerized wall. Grayscale values at a length of 0.03 mm were measured using Image Analyzer software (vers. 1.42.1, MEESOFT).

#### 2.4.2. Evaluation of Benchmark Masters and Replicas

Benchmarks BM1, BM2, and BM3 underwent an evaluation of the designed geometry parameters after the VPP process. The width and height of the ribs were measured using a Nikon SMZ1270 optical microscope, performing 5 measurements for each rib. The values of the width and height of the ribs were expressed as mean ± standard deviation. From these results, the aspect ratios achievable by VPP were quantified as the width/height ratio. Additionally, the minimum feature size in a master produced by VPP was identified.

Benchmarks BM4 and BM5 were analyzed using a Nikon Eclipse LV150N microscope, for the evaluation of (i) the width and height of the ribs of the benchmark master; and (ii) the width and depth of channels of the replica. The point data were post-processed with Mountains Map software using a “remove form” operator with a first-degree polynomial to make the surface planar and a “remove outliers” operator to remove incorrect measurement values. Values of the width and height of ribs and depth of channels were expressed as mean ± standard deviation. In this case, the use of the high magnification of the Nikon Eclipse LV150N microscope was essential due to the small size of the BM4 and BM5 features under analysis.

Furthermore, to obtain an evaluation of the surface roughness of the replicated PDMS device, four measurements were performed both on the surface surrounding the channels and inside the 500 µm-wide channel for the PDMS replica of BM4 using the Nikon Eclipse LV150N profilometer (Nikon, Tokyo, Japan) equipped with a confocal head. Each measurement was performed at a random position, scanning an area of 2000 × 500 µm^2^ (x, y) with a magnification of 200× and a layer thickness of 0.10 µm. The results of the profilometry analysis were obtained by analyzing the 3D point data, which were post-processed using Mountains Map software (Digital Surf, Besançon, France). The post-processing included a “remove form” to make the surface planar and a “remove outliers” operator to remove incorrect measurement values. Values of surface roughness S_a_ were expressed as mean ± standard deviation.

## 3. Results and Discussion

### 3.1. Methodology Validation

The measurements of the height of BM3 ribs using two different methods to evaluate the measurement error are shown in Table 3. The results indicate that the error introduced by both instruments is below the nominal value of the BM3 ribs. Furthermore, it is noteworthy that within the dimensional range of the considered ribs, the error associated with optical microscopy was lower compared to that of profilometry analysis. Therefore, optical microscopy was selected as the method for measuring the ribs of benchmarks BM1, BM2, and BM3 using the SMZ1270 microscope, significantly reducing the analysis time.

**Figure 1:**
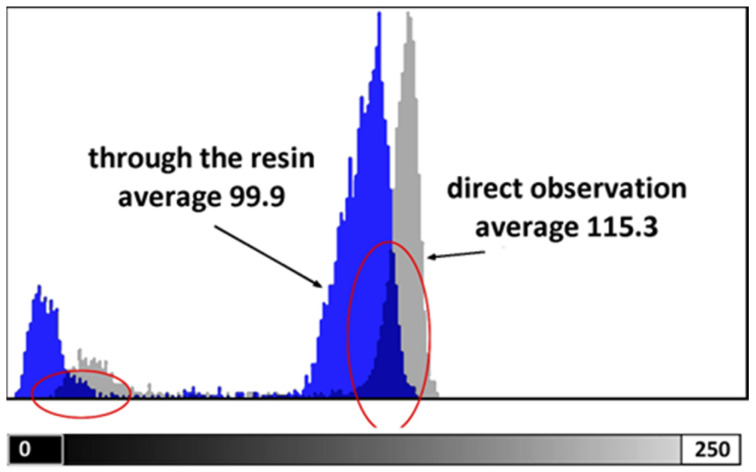
Grayscale distribution is measured on the image of the ruler by direct observation (grey) and through the photopolymerized wall (blue).

The test conducted to assess the loss of readability through the photopolymerized wall yielded the result depicted in Figure 1. The 1 mm-thick resin wall resulted in an overall brightness reduction of approximately 15%, potentially leading to a loss of information in the darkest details of the image (Figure 1).

**Figure 2:**
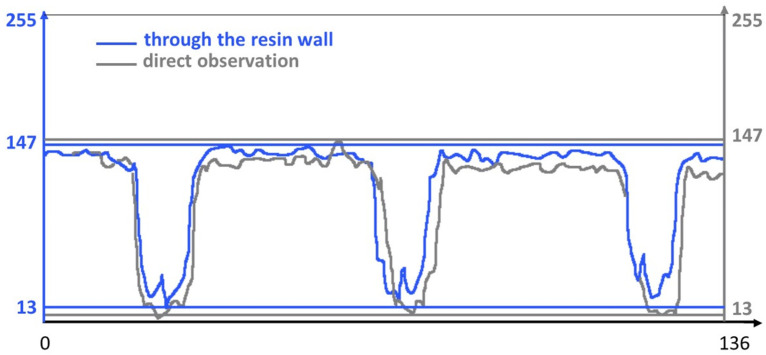
Grayscale values along a 136-pixel line, crossing three marks of the ruler.

Figure 2 illustrates the grayscale values measured by the Image Analyzer software along a line. Despite the slight decrease in brightness, the peaks associated with the image marks remain perfectly distinguishable. The Michelson global contrast index value from Equation (1) was *C* = 0.87 for observation through the resin wall and *C* = 0.91 for direct observation. This indicates a loss in image readability of approximately 7%.

### 3.2. Evaluation of Benchmark Feasibility

#### 3.2.1. Morphology of Benchmark Masters

**Figure 3:**
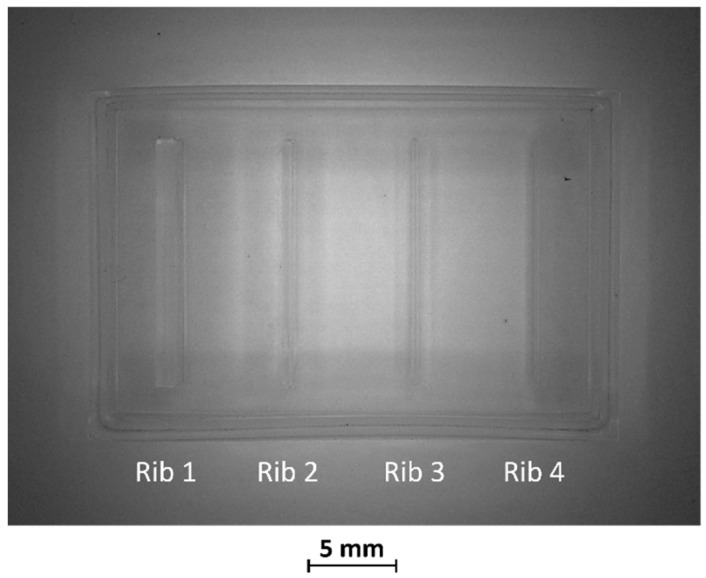
Top view of BM1.

**Figure 4:**
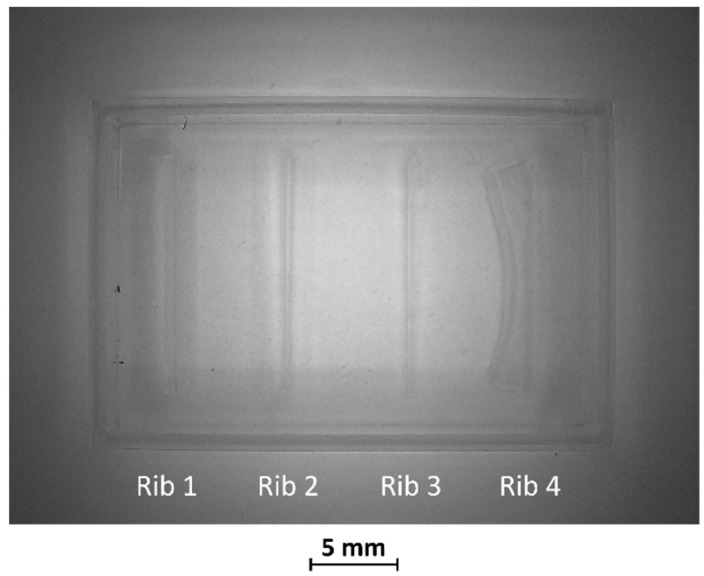
Top view of BM2. Rib 3 and rib 4, with a width/height ratio of 1:19 and 1:60, respectively, were both collapsed.

**Figure 5:**
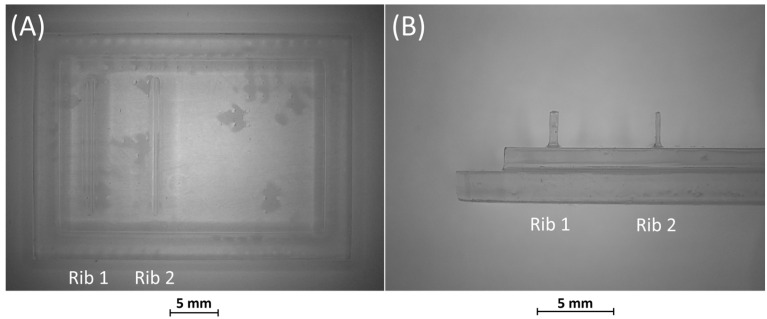
(**A**) Top view and (**B**) lateral view of BM3, fabricated without the surrounding casting box.

After production by VPP, benchmark masters were observed using an optical microscope to evaluate the designed geometry parameters. Optical micrographs of BM1, BM2, and BM3 masters are shown in Figure 3, Figure 4 and Figure 5. The four ribs of BM1 appear well defined (Figure 3).

In BM2, the two thinnest ribs (ribs 3 and 4) collapsed during manufacturing, as visible in Figure 4 Therefore, the fabrication process for ribs designed with an aspect ratio lower than 1:12 failed using the VPP technique with the printing parameters reported in Table 2.

BM3 presents both the ribs accurately produced (Figure 5A,B).

**Figure 6:**
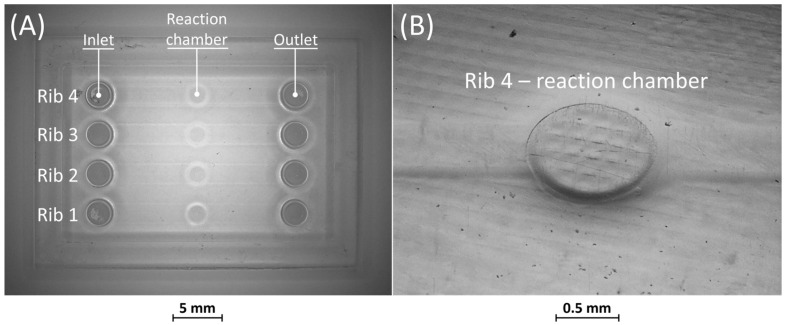
(**A**) Top view of BM4 master with ribs of nominal widths of 50 μm (rib 1), 80 μm (rib 2), 250 μm (rib 3), and 500 μm (rib 4). (**B**) Detail of the reaction chamber of rib 4.

**Figure 7:**
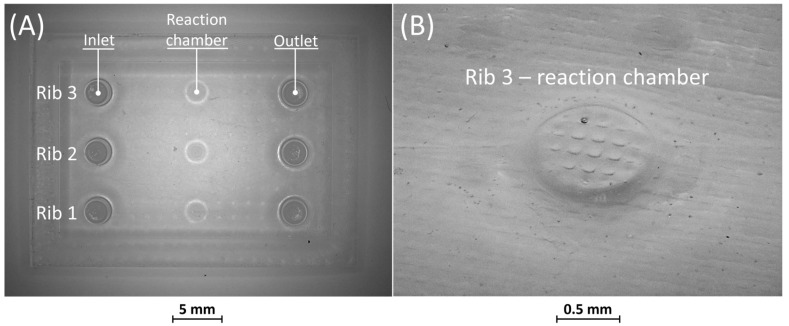
(**A**) Top view of BM5 master with ribs of nominal widths of 10 μm (rib 1), 25 μm (rib 2), and 50 μm (rib 3). (**B**) Detail of the reaction chamber of rib 3.

Optical observations of BM4 and BM5 masters are presented in Figure 6 and Figure 7. All the ribs of BM4 were effectively fabricated, and the reaction chamber was connected to the channels (Figure 6A). The reaction chamber of rib 4 is shown, as an example, in Figure 6B. Inlet and outlet connecting channels in the replica are usually formed manually and reached with biopsy needles. The ability to obtain them directly during the fabrication of the replica, taking advantage of the benefits of VPP technology, means a reduction in human intervention.

Figure 7A shows the optical micrography of BM5, where the replication process successfully reproduced ribs with thicknesses of 25 µm and 50 µm, capturing a sufficient level of detail. The detail of the reaction chamber of rib 3 is presented in Figure 7B, as an example. However, rib 1 of BM5 with a nominal width of 10 µm was not feasible for VPP technology as it fell below the general minimum resolution of the VPP machine in the XY plane, which is about 25 µm [29]. Therefore, BM5 was instrumental in testing the limits of the proposed manufacturing chain by designing extremely narrow ribs.

#### 3.2.2. Morphology of Benchmark Replicas

**Figure 8:**
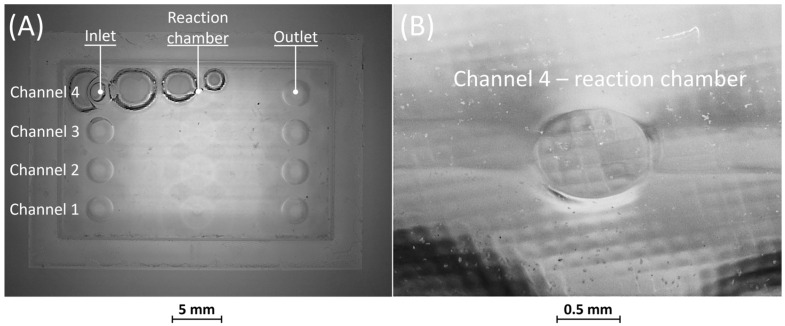
(**A**) Top view of BM4 replica showing defects on the surface opposite to that containing the microfluidic channels. (**B**) Detail of the reaction chamber of rib 4.

**Figure 9:**
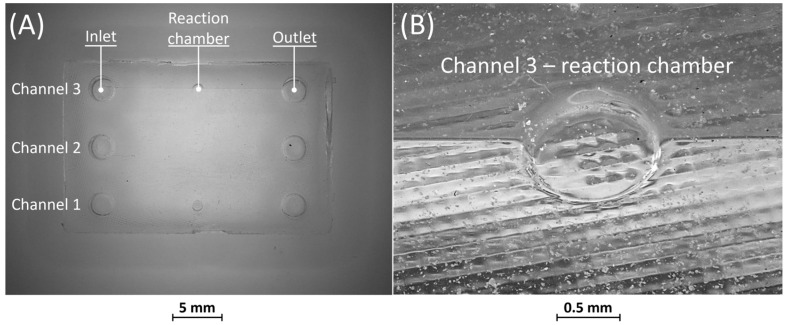
(**A**) Top view of BM5 replica and (**B**) detail of the reaction chamber of rib 3.

Figure 8 and Figure 9 display the corresponding replicas of BM4 and BM5. In both replicas, the inlet and outlet have been accurately reproduced. Figure 8A reveals that the replica of BM4 was not correctly formed during the consolidation phase of the PDMS in the mold containing the master. Nevertheless, this does not impact the proper functioning of the replica, as the defects are on the surface opposite to that containing the microfluidic channels. Figure 8B shows how the microfluidic features, including the channels and the reaction chamber, were accurately reproduced.

No defects were identified in the PDMS casting phase of BM5, as depicted in Figure 9A. Furthermore, similar to BM4, it is evident from Figure 9A,B that all microfluidic features were faithfully reproduced.

Overall, the combination of vat photopolymerization followed by replication demonstrated practicality for channel sizes wider than 25 µm. Additionally, the replicas of BM4 and BM5 confirmed that the removable casting box effectively seals the PDMS during casting, preventing any leakages.

#### 3.2.3. Ribs and Channel Evaluation

**Figure 10:**
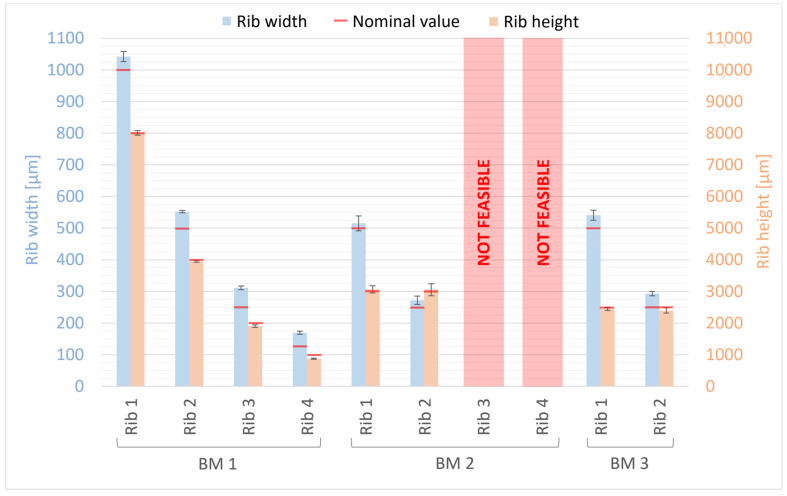
Dimensional evaluation of the width and height of BM1, BM2, and BM3 ribs. The nominal value from the design is reported as a red line.

The width and height measurements of ribs for BM1, BM2, and BM3 are presented in Figure 10. For comparison, nominal values of rib width and height from the design are indicated by a red line. The deviations from the nominal values ranged from +4% to +36% for rib width and from −13% to +2% for rib height (Figure 10). Ribs 3 and 4 of BM2 could not be quantified as they collapsed during the manufacturing process (Figure 4). Generally, the manufactured ribs of BM1, BM2, and BM3 are wider and shorter than the designed ones, with the percentage error increasing as rib size decreases, both for width and height. A higher percentage error was found for the width than for the height of the ribs; however, the deviation is smaller for the width (ranging from +15 μm to +61 μm) than for the height (ranging from −127 μm to +65 μm) of the ribs. Moreover, the measurements of BM3 for the width and height of the ribs confirm that the presence of the casting box has no influence on the dimensional tolerance of the ribs, so that they are fully comparable with BM1 and BM2 as well as with their nominal values from the design.

**Figure 11:**
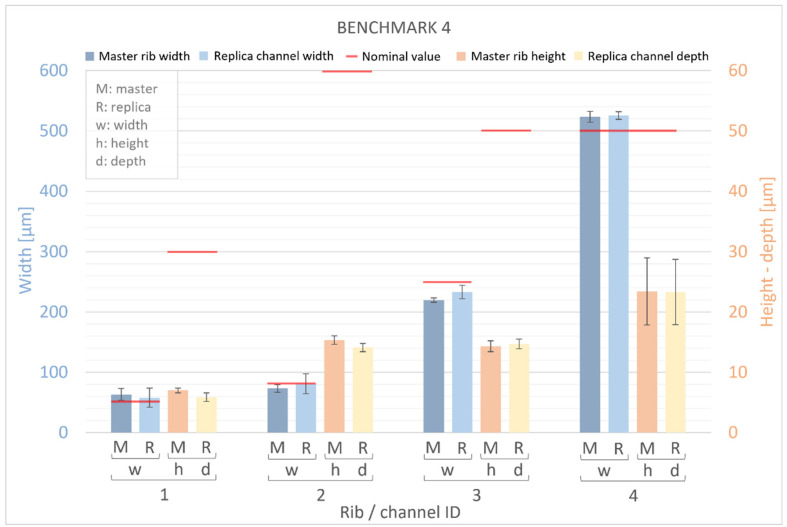
Dimensional evaluation of the width (w) and height (h) of BM4 master (M) ribs and the depth of replica (R) channels. The nominal value from the design is represented by a red line.

**Figure 12:**
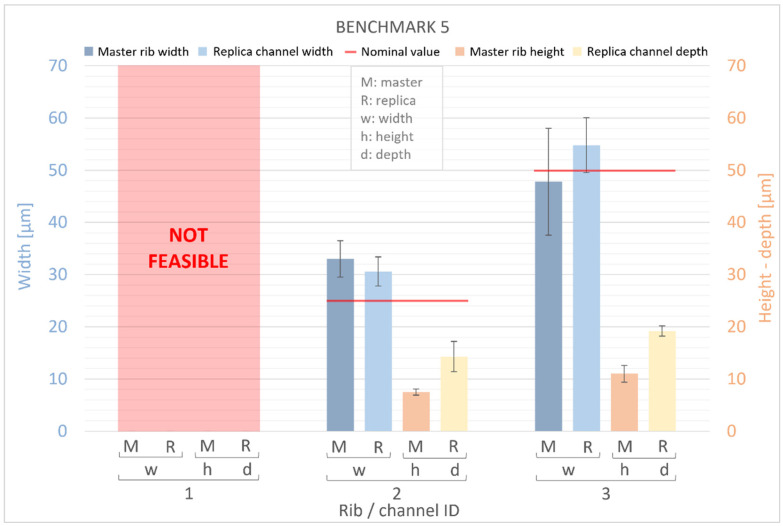
Dimensional evaluation of the width (w) and height (h) of BM5 master (M) ribs and the depth of replica (R) channels. The nominal value from the design is represented by a red line.

Figure 11 and Figure 12 present measurements of the ribs of the BM4 and BM5 masters, along with their corresponding replica channels. Nominal values for the width, height, and depth of both ribs and channels are represented by a red line. For the master benchmarks BM4 and BM5, deviations from the nominal values are observed. The width of the ribs exhibits percentage error deviations ranging from −12% to +32%, while the height shows deviations from −78% to −53%. In BM4 and BM5, similar to BM1–BM3, the manufactured ribs are generally wider and shorter than the designed ones. However, a generally higher percentage error is observed for the height compared to the width, with the width showing a slightly smaller deviation (ranging from −30 μm to +23 μm) than the height (ranging from −45 μm to −18 μm) of the ribs. Furthermore, it is worth noting that there is a significant dimensional error for the width of all ribs below 50 µm and for the height of all ribs below 60 µm. Conversely, the accuracy of the VPP technology is confirmed for thicker features.

The results obtained for the replicas were consistent with those for the master devices, demonstrating that the manufactured channels were generally wider and shorter than the designed channels. The devices exhibited percentage error deviations from the nominal values ranging from −7% to +23% in the width of the channels and from −80% to −53% in the height. Similar to the masters, a generally higher percentage error was found for the height than for the width of the channels, with a slightly lower deviation for the width (ranging from −17 μm to +25 μm) than for the height (ranging from −46 μm to −11 μm) of the channels. It is interesting to note that the replica reproduced the original quite accurately. In fact, the numerical deviation of the replica’s width compared to the original template varies between −6 μm and +15 μm, while for the height it ranges from −1 μm to +8 μm. The percentage error falls within the range of −9% to +15% for width and between −16% and +91% for height, respectively.

Table 4 shows the measured values of the width/height (w/h) ratio of BM1–BM5, emphasizing deviations from their nominal values. It is evident from the obtained w/h values that all ribs and channels were generally wider and shorter than originally designed, as discussed earlier. For BM1, the aspect ratio increases as the rib height decreases, deviating significantly from the nominal values. In contrast, values obtained for BM2 and BM3 are close to the expected ones. The width/height ratio deviates notably from the nominal value for BM4 and BM5, in both masters and replicas. This discrepancy is attributed to the rib and channel height being of the same order of magnitude as the layer thickness used in the VPP process.

Overall, the masters (BM1–BM5) produced by VPP significantly surpass the limits of soft lithography, where the minimum width/height ratio is typically 1:4 [23].

The results obtained from all the master benchmarks revealed variations in both width and height values compared to the nominal values. In terms of absolute average values, the deviation for width is (28 ± 16) μm, while for height it is (51 ± 27) μm. These deviations can be attributed to two distinct effects, both associated with the VPP process.

The error in feature width may arise from the size of the laser spot, which, in this study, is 40 μm. The energy distribution of the laser spot is not uniform but follows the Gaussian type TEM_00_ [43]. To consolidate the resin during the VPP process, the total incident energy along the scan vector must exceed a critical value known as the critical exposure [43]. The energy was supplied over an area smaller than the actual diameter of the laser spot, given the Gaussian distribution causing reduced incident energy at the periphery of the spot. Consequently, the resin could not attain a sufficient energy level to trigger polymerization, potentially leading to a different degree of polymerization in the core and peripheral regions. Therefore, the material beneath the peripheral region of the spot could not fully polymerize towards the outer edge of the rib, while the material facing the filling zone could achieve complete polymerization, thanks to adjacent laser passes in the core region. Additionally, it can be assumed that the unpolymerized material was removed during the washing phase, resulting in errors in the XY plane smaller than the diameter of the laser spot. Specifically, ribs with a width exceeding than 50 μm, produced by multiple laser passes, exhibited a mean absolute deviation of (35 ± 17) μm, while ribs with a width below 50 μm, characterized by a single laser pass, showed a deviation of (8 ± 6) μm.

Regarding the height analysis, the results indicate a notable increase in the error with respect to the nominal value as the rib height decreases. The error ranged from a −13% to a substantial −80%. This phenomenon was observed when the height of the rib approached values close to the layer thickness used in the VPP process.

Finally, the results of surface roughness S_a_ for the PDMS replica of BM4 showed a value of (0.60 ± 0.07) μm regarding the surface surrounding the channels and (0.88 ± 0.20) μm inside the 500 μm-wide channel.

## 4. Conclusions

This experimental study evaluates the application of vat photopolymerization (VPP) for manufacturing masters used in microfluidic devices. The feasibility of this technology was assessed employing benchmarks to reduce the steps required to produce PDMS replicas. Master benchmarks (BMs) were designed considering parameters such as rib width, height, and the relative width/height ratio (BM1–5). In addition, ribs were connected to the inlet, outlet, and the reaction chamber of the master in BM4 and BM5, whose PDMS replicas were produced by casting. An extensive dimensional characterization was conducted on BM and replicas, using an optical technique based on the Michelson global contrast index. The main results obtained in this study can be summarized as follows:

- The optical measurement technique was successfully validated, with negligible loss of readability. This was expressed as an overall brightness attenuation of about 15% and a loss of approximately 7% in the Michelson global contrast index;
- The optical tests also confirmed the feasibility of a hybrid solution (BM3) where the casting box is built separately from the master, enabling direct PDMS casting without additional steps;
- The feasibility of a hybrid solution (BM3) was also verified by the fact that the casting box has no influence on the dimensional tolerance of the ribs;
- VPP technology allows the production of master benchmarks (BM1–BM5) with ribs having a minimum thickness of 25 µm and an aspect ratio of 1:12, overcoming the dimensional limitations of soft lithography;
- Ribs in BM1–3 were generally wider and shorter than designed ones, exhibiting a maximum deviation of +36% in width and −13% in height;
- Benchmarks BM4 and BM5 showed percentage error deviations from the nominal values, with a maximum +32% in the width of the ribs and −78% in the height of the ribs. This corresponds to a height deviation of 27 µm, approximately equivalent to the VPP layer thickness;
- The width and height values of all the master benchmarks display variations from the nominal values due to the VPP process. These variations offer valuable insights into the orientation of parts for additive manufacturing;
- All microfluidic features of BM4 and BM5 were faithfully reproduced by PDMS replicas, with numerical deviations from masters varying between −6 μm and +15 μm for width and between −1 μm and +8 μm for height.

The results obtained in this study demonstrated the suitability of vat photopolymerization followed by replication for manufacturing specifically designed microfluidic devices. This approach enhances the design limitations and mitigates the multi-step procedures associated with conventional soft lithography.

## Figures and Tables

**Table 1:** Design and geometrical parameters of benchmark (BM) masters: rib width (w); rib height (h); and width/height ratio (w/h) based on the rib width (µm) and height (µm).

BM1												
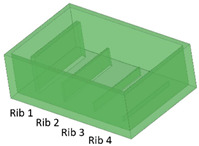	Rib 1			Rib 2			Rib 3			Rib 4		
	w (µm)	h (µm)	w/h	w (µm)	h (µm)	w/h	w (µm)	h (µm)	w/h	w (µm)	h (µm)	w/h
	1000	8000	1:8	500	4000	1:8	250	2000	1:8	125	1000	1:8
BM2												
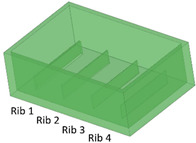	Rib 1			Rib 2			Rib 3			Rib 4		
	w (µm)	h (µm)	w/h	w (µm)	h (µm)	w/h	w (µm)	h (µm)	w/h	w (µm)	h (µm)	w/h
	500	3000	1:6	250	3000	1:12	160	3000	1:19	125	3000	1:24
BM3												
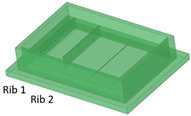	Rib 1			Rib 2			-					
	w (µm)	h (µm)	w/h	w (µm)	h (µm)	w/h						
	500	2500	1:5	250	2500	1:10						
BM4												
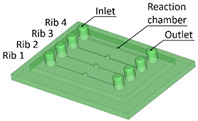	Rib 1			Rib 2			Rib 3			Rib 4		
	w (µm)	h (µm)	w/h	w (µm)	h (µm)	w/h	w (µm)	h (µm)	w/h	w (µm)	h (µm)	w/h
	50	30	1.7:1	80	60	1.3:1	250	50	5:1	500	50	10:1
BM5												
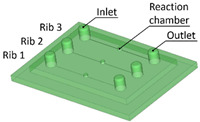	Rib 1			Rib 2			Rib 3			-		
	w (µm)	h (µm)	w/h	w (µm)	h (µm)	w/h	w (µm)	h (µm)	w/h			
	10	10	1:1	25	25	1:1	50	50	1:1			

**Table 2:** Process parameters used to produce the benchmark masters by VPP.

Process Parameter	Unit	Result
Contours	(n)	3
Hatch distance	(mm)	0.5
Laser speed	(mm/min)	5000
Layer thickness	(µm)	30
Laser spot	(µm)	40
Laser wavelength	(nm)	405

**Table 3:** Results of the height measurements of BM3 ribs, carried out by optical microscopy and profilometry analysis.

Rib ID	Nominal Value (μm)	Optical Microscopy		Profilometry	
		(Average ± St. Dev.) (μm)	Error (%)	(Average ± St. Dev.) (μm)	Error (%)
Rib 1	2500	(2450 ± 52)	−2	(2296 ± 94)	−8
Rib 2	2500	(2397 ± 83)	−4	(2309 ± 69)	−8

**Table 4:** Width/height (w/h) ratio values measured for all the BM masters and replicas.

BM ID	BM Type	Rib/Channel ID	Nominal w/h Ratio	Measured w/h Ratio
BM1	Master	Rib 1	1:8	1:7.7
		Rib 2	1:8	1:7.2
		Rib 3	1:8	1:6.1
		Rib 4	1:8	1:5.2
BM2	Master	Rib 1	1:6	1:6.0
		Rib 2	1:12	1:11.2
		Rib 3	1:19	Not feasible
		Rib 4	1:24	Not feasible
BM3	Master	Rib 1	1:5	1:4.5
		Rib 2	1:10	1:8.2
BM4	Master	Rib 1	1.7:1	9.1:1
		Rib 2	1.3:1	4.8:1
		Rib 3	5:1	15.3:1
		Rib 4	10:1	22.4:1
BM5	Master	Rib 1	1:1	Not feasible
		Rib 2	1:1	4.4:1
		Rib 3	1:1	4.3:1
BM4	Replica	Channel 1	1.7:1	9.8:1
		Channel 2	1.3:1	5.8:1
		Channel 3	5:1	15.9:1
		Channel 4	10:1	22.5:1
BM5	Replica	Channel 1	1:1	Not feasible
		Channel 2	1:1	2.1:1
		Channel 3	1:1	2.9:1

## Data Availability

The data are available upon request.

## Supplementary Materials

The following supporting information can be downloaded at: https://www.mdpi.com/article/10.3390/bioengineering11010080/s1, Table S1: Nominal characteristics of acrylic acid ester employed for the benchmark masters’ production; Table S2: Properties of PDMS used to produce replicas of BM4 and BM5.
