# Supplementary material for: Features of Vat-Photopolymerized Masters for Microfluidic Device Manufacturing
Source: Bioengineering (Basel). 2024 Jan 15;11(1):80. doi: 10.3390/bioengineering11010080 (PMC10813418; doi:10.3390/bioengineering11010080)
Supplement: Supplementary file 1 [file bioengineering-11-00080-s001.zip › bioengineering-2788885-supplementary.pdf]

Table S1. Nominal characteristics of acrylic acid ester employed for the benchmark masters' production [40].

Property	Unit	Result (min ÷ max)
Tensile modulus of elasticity	[MPa]	1700 ÷ 2200
Tensile strength	[MPa]	45 ÷ 55
Elongation at break	[%]	6 ÷ 10
Flexural modulus of elasticity	[MPa]	2000 ÷ 2500
Viscosity at 25°C (before photopolymerization)	[mPa*s]	1000 ÷ 1400
Density (before photopolymerization)	[g/cm^3]	1.01

Table S2. Properties of PDMS used to produce replicas of BM4 and BM5 [41].

Property	Unit	Result
One or two part		Two
Color		Colorless
Viscosity (base)	[mPa*s]	5100
Viscosity (mixed)	[mPa*s]	3500
Thermal conductivity	[W/m*K]	0.27
Specific gravity (cured)		1.03
Pot life at 25 °C	[h]	1.5
Cure time at 25 °C	[h]	48
Heat cure time at 100 °C	[min]	35
Heat cure time at 125 °C	[min]	20
Heat cure time at 150 °C	[min]	10
Hardness	[shore A]	43
Dielectric strength	[kV/mm]	19
Volume resistivity	[ohm*cm]	$2.9*10^{14}$
Tensile strength	[MPa]	6.7
Refractive index at 589 nm		1.4118
Refractive index at 632.8 nm		1.4225
Refractive index at 1321 nm		1.4028
Refractive index at 1554 nm		1.3997

[40] DWS Brochure - Vitra DL375. <https://www.dwssystems.com/it/configurator/X/2/vitra-dl375> Accessed 24.1.11.

[41] Dow - SYLGARD™ 184 Silicone Elastomer Technical Data Sheet. < https://www.dow.com/content/dam/dcc/documents/en-us/productdatasheet/11/11-31/11-3184-sylgard-184-elastomer.pdf?iframe=true> Accessed 24.1.11.
